# Human-machine-human interaction in motor control and rehabilitation: a review

**DOI:** 10.1186/s12984-021-00974-5

**Published:** 2021-12-27

**Authors:** Emek Barış Küçüktabak, Sangjoon J. Kim, Yue Wen, Kevin Lynch, Jose L. Pons

**Affiliations:** 1 Department of Mechanical Engineering, McCormick School of Engineering, Northwestern University, 60208 Evanston, IL USA; 2 Legs + Walking Lab, Shirley Ryan Ability Lab, 60611 Chicago, IL USA; 3 Department of Physical Medicine and Rehabilitation, Feinberg School of Medicine, Northwestern University, 60611 Chicago, IL USA; 4 Department of Biomedical Engineering, McCormick School of Engineering, Northwestern University, 60208 Evanston, IL USA

**Keywords:** Human-machine-human interaction, Human-robot interaction, Multi-human interaction, Motor control, Rehabilitation, Motor learning

## Abstract

**Background:**

Human-human (HH) interaction mediated by machines (e.g., robots or passive sensorized devices), which we call human-machine-human (HMH) interaction, has been studied with increasing interest in the last decade. The use of machines allows the implementation of different forms of audiovisual and/or physical interaction in dyadic tasks. HMH interaction between two partners can improve the dyad’s ability to accomplish a joint motor task (*task performance*) beyond either partner’s ability to perform the task solo. It can also be used to more efficiently train an individual to improve their solo task performance (*individual motor learning*). We review recent research on the impact of HMH interaction on task performance and individual motor learning in the context of motor control and rehabilitation, and we propose future research directions in this area.

**Methods:**

A systematic search was performed on the Scopus, IEEE Xplore, and PubMed databases. The search query was designed to find studies that involve HMH interaction in motor control and rehabilitation settings. Studies that do not investigate the effect of changing the interaction conditions were filtered out. Thirty-one studies met our inclusion criteria and were used in the qualitative synthesis.

**Results:**

Studies are analyzed based on their results related to the effects of interaction type (e.g., audiovisual communication and/or physical interaction), interaction mode (collaborative, cooperative, co-active, and competitive), and partner characteristics. Visuo-physical interaction generally results in better dyadic task performance than visual interaction alone. In cases where the physical interaction between humans is described by a spring, there are conflicting results as to the effect of the stiffness of the spring. In terms of partner characteristics, having a more skilled partner improves dyadic task performance more than having a less skilled partner. However, conflicting results were observed in terms of individual motor learning.

**Conclusions:**

Although it is difficult to draw clear conclusions as to which interaction type, mode, or partner characteristic may lead to optimal task performance or individual motor learning, these results show the possibility for improved outcomes through HMH interaction. Future work that focuses on selecting the optimal personalized interaction conditions and exploring their impact on rehabilitation settings may facilitate the transition of HMH training protocols to clinical implementations.

**Supplementary Information:**

The online version contains supplementary material available at 10.1186/s12984-021-00974-5.

## Introduction

### Human-human interaction

Humans often interact with each other while performing motor tasks, either to improve performance by working as a group or to learn from each other. Some motor tasks, such as exercising an injured joint, can be accomplished by a single person, but also allow two or more people (e.g., the therapist and the patient) to interact during performance of the task. Other motor tasks require multiple people to interact and cannot be performed by a single person (e.g., carrying a large table). Human-human (HH) interaction in tasks such as these can take the form of audiovisual communication and/or physical interaction.

In recent years, many studies have compared motor task performance under solo conditions and under different types of interaction among groups of two or more [1–5]. For tasks that can be performed with one or more people, we refer to task performance as the individual ability to accomplish a motor task during or without interaction. Similarly, for tasks that require multiple people, task performance is the ability to perform the task as a group.

There has been an increasing interest also for the effects of HH interaction on individual motor learning [2, 6–8] for motor tasks. In this article, we refer to individual motor learning as the solo task performance difference before and after a training period. This training phase can be performed individually or by interacting with someone else. In this review, we are interested in the learning of only individuals but not groups. Therefore, we do not analyze motor learning for tasks that require multiple people.

Studies that have demonstrated better outcomes of HH interaction than solo performance or training often attributed these positive effects to (1) increased motivation and interest [3, 9, 10]; (2) the ability to estimate partner’s motion, [11, 12]; and (3) the summing of physical effort, or the ability of partners to communicate and adopt specialized roles in performing a physical task [1, 13].

Even though important results have been obtained showing the potential benefits of human-human interaction, it is unclear how different kinds of interactions impact task performance and individual motor learning. A better understanding of how humans interact with each other in motor control tasks will have a significant impact on rehabilitation robotics, industrial collaborative robotics, and social robotics.

### Robots as a medium for human-human interaction

Conventional human-human (HH) interaction often occurs through direct physical connection in motor control and rehabilitation contexts. For example, in physical therapy, the therapist interacts with patients by holding their limb to guide or assist them. In some cases, however, it is beneficial to controllably customize the physical interface between the therapist and the patient [14]. One way to achieve this customized interface is to connect each human to a robot. The robots can then be controlled to implement desired interaction dynamics between the humans [2, 15], and possibly to display a virtual external environment [6, 16]. The simplest scenario involves two humans interacting through two robots, but other interaction network topologies, with more humans involved [12] (e.g., for group training), are possible. We call all such systems of robot-mediated interacting humans “human-robot-robot-human” (HRRH) interaction, or “human-robot-human” (HRH) for short.

In addition to physical interaction, HRH systems support non-physical interaction (e.g., audiovisual interaction), which can lead to improved motivation and task performance [17–19]. For example, an arm exoskeleton can measure the joint angles of the human arm and estimate the hand’s position. The individuals’ state information can then be projected to a virtual environment where both users see visual cues about themselves and their partners [20]. While these kinds of virtual environments directly provide visual interaction between individuals, they can also indirectly improve auditory interaction by providing more content to talk about with their partner [19, 21].

It is also possible to use passive mechanical devices to transmit physical information between humans, or sensors to collect information for transmission between humans [5, 9, 22–24]. These devices cannot be referred to as robots. To include both robot-mediated and passive-device-mediated systems, we use the term human-machine-human (HMH) interaction.

### Definitions

Throughout this review, we refer to the previously-defined terms “task performance” and “individual motor learning” to describe the goals of particular HMH interactions. It is also worth distinguishing the two main forms of motor learning [25]: motor adaptation is the “*learner’s incremental return to baseline performance in response to an environmental perturbation that causes performance errors*” [26], while skill learning is improving the performance beyond baseline levels in the absence of environmental disturbances [27]. We also use the following terms to categorize the different studies in HMH interaction:

*Interaction type* The interaction type refers to physical interaction, non-physical interaction, or a combination of physical and non-physical interaction. Non-physical interaction includes cases where subjects can see the motion of their partner or partner’s avatar (visual) or can hear their partner (auditory). We also refer to the *interaction characteristics* that characterize the particular interaction type. For example, a physical interaction defined by a virtual spring between two humans is characterized by the stiffness of the spring. In this case, the stiffness is the interaction characteristic, which influences task performance in multi-human interaction [2, 15, 16, 28, 29].

**Fig. 1:**
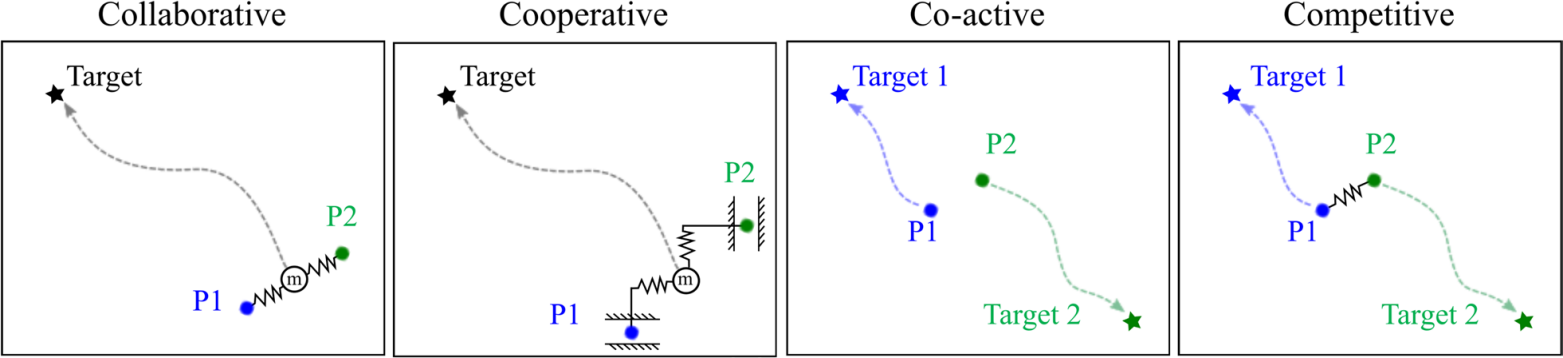
Examples of different interaction modes

*Interaction mode* Figure 1 illustrates the following definitions of interaction modes which have been adapted from Sawers et al. [30] and Jarrasse et al. [31]:

- *Collaborative* Partners share the same task goal and work together to achieve it. Their roles are not assigned a priori.
- *Cooperative* Partners share the same task goal but are assigned to different roles (e.g., teacher and student).
- *Co-active* The task is divisible, and each human works independently, but they still interact.
- *Competitive* Each human tries to achieve their own goal, which is in conflict with the other(s) achieving theirs.

The choice of interaction mode has a significant impact on task performance and engagement [3, 5, 32, 33]. It is worth noting that the term **interaction behavior** is also used in the literature in place of interaction mode [30, 31].

*Partner characteristics* The relative skill levels of interacting partners, the relationship between the partners (e.g., strangers, friends), and the partner’s personality (e.g., extroversion, agreeableness) are referred to as partner characteristics, which have been shown to be important factors influencing task performance and individual motor learning [2, 3, 6–8, 12, 22, 28, 33].

*Interaction condition* The term interaction condition for a particular task refers to one or more of the interaction type, interaction mode, and partner characteristics.

### Contribution of this review

Baur et al. [34] conducted a systematic review to analyze whether multiplayer games improve experience and performance in robot-assisted and virtual reality-assisted rehabilitation. They mainly focused on studies with non-physical interaction and compared the methods and results of papers with different game modes. Sawers et al. [30] performed a comprehensive review with a focus on the physical interaction between humans with consideration to the perspective on rehabilitation robotics. Moreover, they provided detailed taxonomies of human-human sensorimotor interaction.

Due to the complex nature of human-human interaction, the effects of interaction conditions on task performance and individual motor learning are not well understood. Also, considering the large amount of recent work, we believe a broader and more up-to-date review with a focus on the effects of different interaction conditions is needed. In this review, we provide a comprehensive analysis of experimental designs and results by covering the interaction conditions defined above. We address the following research question: How does human interaction in HH and HMH systems influence task performance and individual motor learning? We conducted a systematic literature search focused on studies that investigate the effects of interaction conditions on coordinated work between humans mediated by machines. The different experimental methods and performance metrics of these studies were identified.

The core of this paper analyzes the results of the selected studies according to the main factors influencing the HMH interaction: interaction type, interaction mode, and partner characteristics. While we highlight consistent results in these references, we also examine conflicting outcomes between different studies. This review also identifies gaps in the literature and indicates directions for future research.

### Outline

We first present the details of our search criteria and statistics of the study selection process under the Methods section. Then we provide a comprehensive analysis of the experimental design and methods used only in the selected references. This section presents different experimental setups, their implementation details, and metrics used in the selected studies. In the Results section, we focus on the findings of the selected studies related to the effects of the interaction conditions on task performance and individual motor learning under three main categories: (1) interaction type and characteristics, (2) interaction mode, and (3) partner characteristics. In the Discussion, we analyze the experimental designs and results of the selected papers with reference to papers that were excluded from the core analysis. We also highlight conflicting and consistent results in the literature and suggest directions for future research.

## Methods

A comprehensive review of the literature to synthesize the effects of HH and HMH interaction in motor control and rehabilitation has been conducted. The review methodology is described in following subsections.

### Search criteria

An electronic search on Scopus, IEEE Xplore, and PubMed databases was conducted. The search was limited to literature written in English. No restriction on the publication date was considered. Both conference papers and journal articles were included. We finalized the search in April 2021.

The search query was designed such that the resulting studies have the following characteristics: (1) they involve robotic or related devices, (2) they include tasks related to rehabilitation or motor control, (3) they involve multiple subjects interacting with each other, and (4) they refer to physical and/or non-physical interaction. Keywords used for each criterion are presented in Table 1. Exact search queries for each database can be seen in Additional file 1.

**Table 1:** Search criteria and simplified version of the phrases used in the systematic search

Criteria	Search phrase
Involves robotic or related devices	(Exoskeleton OR robot)
AND	AND
Studies about rehabilitation or motor control	(Rehabilitation OR therapy OR “motor (control OR learning)”)
AND	AND
Involves multi-human interaction	(Pairs OR dyads OR multiplayer OR telerehabilitation)
AND	AND
Refers to physical or non-physical interaction	(“(Haptic OR physical OR social) interaction” OR motivation OR game)

The search resulted in 863 papers: 614 papers from Scopus, 208 papers from IEEE Xplore, and 41 papers from PubMed.

### Study selection

**Fig. 2:**
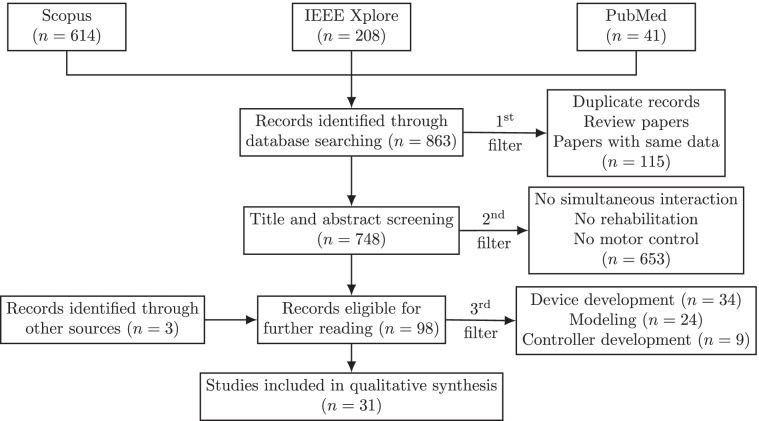
Flowchart of study selection (PRISMA diagram [35])

We filtered the studies using three main steps to eliminate papers that were not related to the focus of this review. The methodology is presented in Fig. 2 as a PRISMA diagram [35].

First, duplicate records from different databases, review papers, position papers, and conference papers presenting the same data with a journal article were filtered out. Second, papers were excluded unless (1) they included simultaneous interaction between at least two people and (2) the tasks were related to physical rehabilitation or motor control. For example, telerehabilitation systems where the therapist can only monitor the status of the patient, studies where there is only human-robot interaction, work related to social therapy, and studies with non-human subjects were excluded. Third, only studies that investigated the effects of interaction type (e.g., physical interaction vs. visual interaction), interaction mode (e.g., cooperative vs. competitive), or partner’s characteristics (e.g., novice vs. expert) in motor control or rehabilitation were included. Studies focusing on modeling human motion and interaction force, or on development of multi human-robot systems and controller design, were excluded at this step. Even though one of our search criteria includes keywords such as “robot” and “exoskeleton,” we did not filter out studies that used only sensory systems due to their relevance to human-robot systems.

Out of 863 papers, thirty-one papers were found to satisfy the inclusion criteria.

### Data extraction

**Fig. 3:**
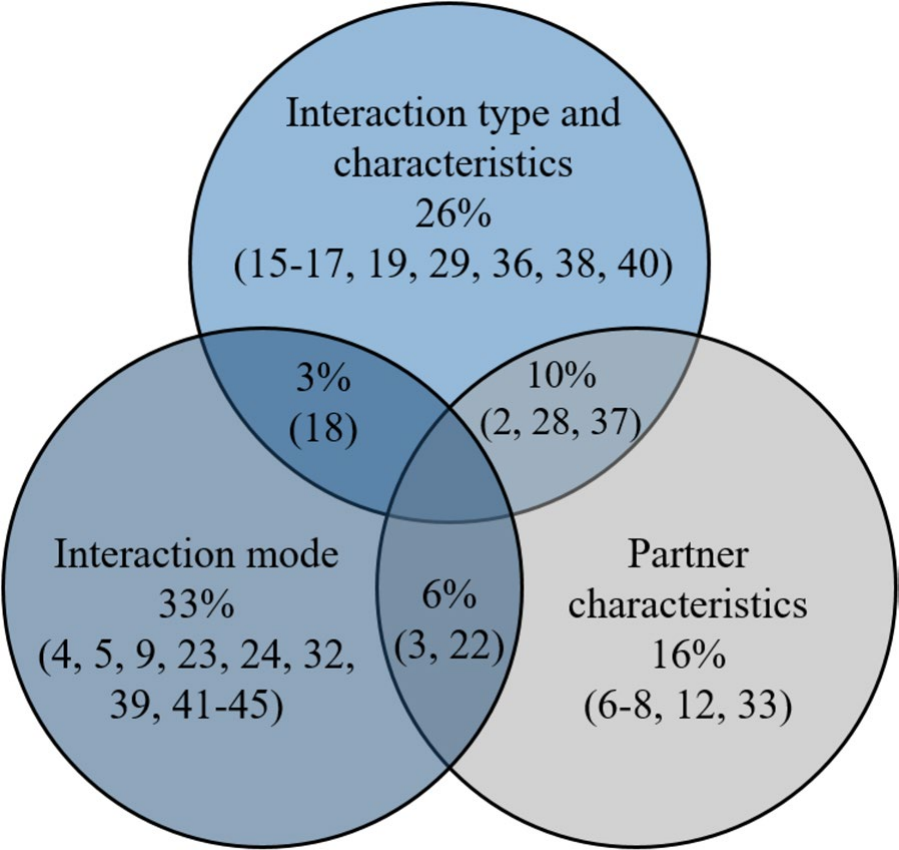
The focus of the research question in selected studies

Data extraction and systematic synthesis were performed on the included studies. First, these papers were categorized into three groups based on their primary research topic: (1) the effect of the interaction type (i.e., physical, non-physical, combination) or characteristics (i.e., spring stiffness, auditory, visual); (2) the effect of the interaction mode (i.e., cooperative, collaborative, co-active, competitive); and (3) the effect of the partner’s characteristics (e.g., novice, experienced, healthy, impaired). Categories of the selected studies are presented in Fig. 3. Articles investigating more than one of the three research topics above appear in each relevant group.

We analyzed each paper in detail by extracting the following information: experimental task, measured variables, focus of the research question, interaction type and its characteristics, interaction mode, partner characteristics and presented results.

## Experimental design and methods

This section describes the experimental design and methods used in the selected studies. This was done in terms of (1) the tasks chosen for coordinated HMH interaction, (2) the way interaction forces were rendered between humans, and (3) the measurements used to assess the experimental outcomes.

### Experimental tasks

**Fig. 4:**
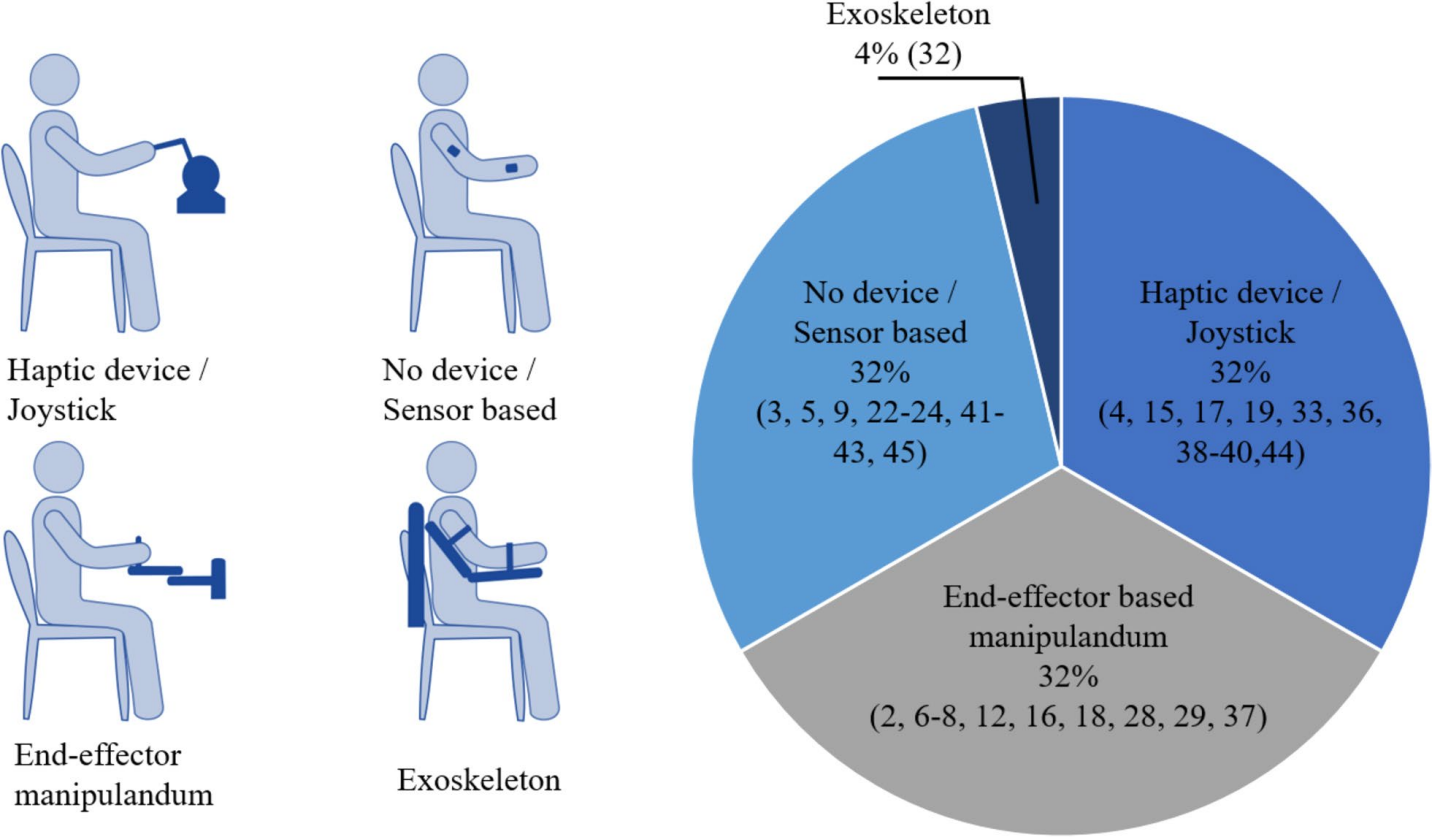
Devices used in the selected studies

**Fig. 5:**
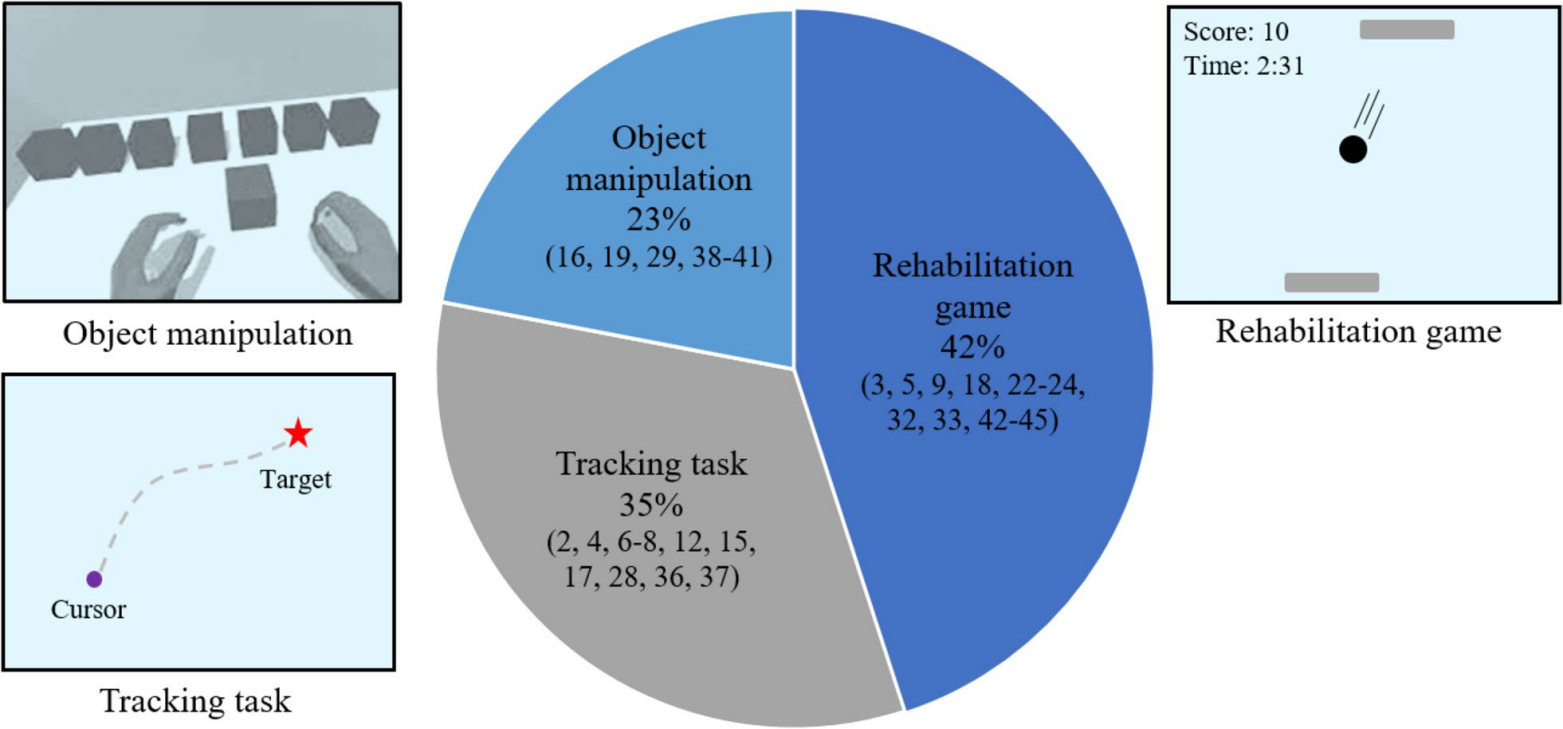
Experimental tasks used in the selected studies

The devices and experimental tasks used in the selected studies are shown in Figs. 4 and 5. A more detailed summary of the experimental tasks and devices used in each study are given in Additional file 2: Table S1.

#### Tracking tasks

Eleven studies investigated the effect of dyadic interaction in planar (2D) arm tracking tasks [2, 4, 6–8, 12, 15, 17, 28, 36, 37]. These studies were based on moving a cursor to a moving [2, 4, 7, 12, 15, 37] or a stationary [6, 17, 28] target, following a path with a cursor  [8], and maneuvering a virtual object on a confined path while trying to prevent collision with the environment [36]. Hand-held end-effector-type manipulators or haptic devices were used as input devices for moving the cursors or virtual objects. The cursors or virtual masses controlled by multiple users were virtually coupled.

#### Object manipulation

Seven studies focused on different kinds of object manipulation (e.g., stacking cubes, grasping cylindrical objects, applying specific forces to static tools moving pendulum) with hand-held end-effector type manipulators, haptic devices, or sensorized objects [16, 19, 29, 38–41]. Among them, five studies used a virtual environment (VR) [16, 19, 29, 39, 40] while in the other two studies subjects applied force on a robotic tool [38] , or to a rigid pole [41] without any feedback from a virtual environment. Two of these studies were built on specific applications, including performing a biopsy [40] and assessing spasticity of hypertonic arms [29].

#### Rehabilitation games

Many studies are based on custom-developed rehabilitation games [3, 5, 9, 18, 22–24, 32, 33, 42–45]. Arm rehabilitation systems [3, 9, 18, 22–24, 32, 42, 45], hand rehabilitation systems [33], commercially-available exergaming input devices [5, 44], and touch-screen tablets and conductive handles [43] were used as the input devices for the games. For most of these studies, one participant used a haptic manipulator or rehabilitation system (generally the impaired participant), while others used a commercially-available joystick or mouse. This was to consider the practical deployment and application in real-life rehabilitation scenarios (e.g., telerehabilitation and patient training with a PT or a healthy partner).

### Haptic rendering

There are mainly two types of physical interaction in HRH systems. The first is obtained by rendering passive elements such as a spring and a damper between subjects. The other is obtained by mirroring the force or position between users. Eighteen of the selected studies implemented physical interaction between subjects. While most of them rendered virtual passive elements between the users to investigate the effects of dyadic interaction, a real sensorized rigid object was used in one study [41].

Haptic virtual elements were implemented in task space between the connection points of each user with the robot [2, 4, 6–8, 12, 16, 17, 19, 28, 29, 36, 37, 39, 40] or at the joint level between the joints of users [15]. While some studies used commercial products and their built-in controllers [12, 17, 19, 28, 36, 39, 40], others used their own custom robotic devices and controllers [2, 6–8, 15, 16, 29, 38].

### Metrics

The metrics used to quantify task performance, individual motor learning, motivation, and exercise intensity are listed in Additional file 2: Table S1. This section briefly summarizes some of the different metrics employed.

#### Task performance

Task performance metrics include task completion time [19, 39, 40, 46], success ratio [16, 29], and number of unwanted collisions with the environment [36, 40]. In static target-reaching tasks, completion time (i.e., the time spent to reach the target) was a typical metric [17, 28]. In tasks involving tracking a trajectory, the root-mean-squared or mean error between the actual trajectory and the target trajectory was typically measured [2, 4, 7, 11, 12, 15, 17, 37].

Studies that implemented rehabilitation games mostly used maximum achievable difficulty level of the game [22, 23] or points collected during the game [33, 44] as a measure of the task performance.

For tasks that allow one or more people, task performance difference between a specific interaction condition and solo mode was calculated as the difference between the individual performance during interaction and the succeeding solo performance without any interaction [2, 37]. For tasks that require multiple people, average dyadic performances of different interaction conditions were compared [16, 17].

#### Individual motor learning

Individual motor learning is quantified by the difference between initial solo performance before training and final solo performance after training [2, 7, 8]. For example, participants get baseline test scores (e.g., task completion time, tracking error) during solo performance; then they practice under different dyadic interaction conditions; then they test again under solo conditions. We do not analyze motor learning results on tasks that require multiple people, as team learning is out of the scope of this review.

#### Motivation and exercise intensity

Increasing motivation, engagement, and exercise intensity are important intermediate goals in motor control and rehabilitation contexts and are helpful for achieving improved task performance and individual motor learning. Both objective and subjective metrics are used to measure these factors.

Subjective results can be derived from Intrinsic Motivation Inventory (IMI) questionnaires [5, 9, 18, 19, 22, 24, 32] (measuring enjoyment/interest, effort/importance, perceived competence, and pressure/tension) and other game experience questionnaires [32, 47]. Objective measurements include exercise intensity quantified by the RMS or the mean absolute value of the arm velocity [3, 9, 22, 24] and time spent on the game [3, 5].

## Results

### Effects of the interaction type and characteristics

We grouped the interaction types into three categories: (1) physical interaction, (2) non-physical interaction, and (3) the combination of physical and non-physical interaction. Physical interaction is usually obtained by rendering a spring/damper system between subjects via robotic devices [2, 7, 16, 20, 28]. Non-physical interaction includes auditory or visual interaction [18, 19]. In some cases, both physical and non-physical interactions occur [17, 36, 38, 40]. Characteristics of the interaction are related to the properties of the virtual rendered elements (e.g., spring stiffness, damping ratio) in physical interaction and the communication channels (e.g., visual, auditory, audio-visual) in non-physical interaction.

Twelve studies showed the effects of different interaction types or interaction characteristics [2, 15–19, 28, 29, 36–38, 40]. Five out of the twelve studies analyze the effects of the interaction type [17, 19, 36, 38, 40], six study the characteristics of physical interaction (i.e., properties of the haptic environment) [2, 15, 16, 28, 29, 37], and one studies the characteristics of non-physical interaction (e.g., auditory vs audio-visual) [18]. Details of these studies and the summary of their results can be found in Additional file 2: Tables S2 and S3.

#### Visuo-physical interaction results in better task performance than visual interaction alone

Chellali et al. [40] and Liu et al. [36] compared the cooperative task performance of dyads having only visual interaction with dyads having both visual and physical interaction. While dyads in the study of Liu et al. [36] worked on moving a virtual sphere inside a pipe, dyads in the study of Chellali et al. [40] worked on a virtual biopsy task. These two tasks, performed with haptic devices, are similar in the sense that both reward finishing the task as fast as possible with minimal contact with the environment (pipe or organs). In Liu et al. [36], subjects control the position of the virtual sphere along different axes while they are connected to the sphere with spring and dampers; in Chellali et al. [40], one subject supervises his/her partner through the rigid haptic link between them.

Both studies found that dyadic performance with visuo-physical interaction had less undesired contact with the environment and achieved shorter completion time than the users with only visual interaction. In addition to the better dyadic task performance, the subjective assessment showed the preference of visuo-physical interaction over visual interaction [36].

#### The characteristics of the physical interaction affect task performance and individual motor learning

The effects of different interaction stiffnesses were investigated by five studies [2, 15, 28, 29, 37], and one study analyzed the effect of viscosity and damping ratio [16].

Ganesh et al. [2], Takagi et al. [15], Beckers et al. [37], and Che et al. [28] implemented collaborative interaction where subjects try to move their own cursor to either a dynamic [2, 15, 37] or a static [28] common target. Experimentation was done solo and with subjects’ end effectors haptically connected via virtual springs. In all of these studies, the tracking performance difference between the dyadic and solo trials with different interaction stiffnesses was analyzed to see the effect on the task performance. Moreover, Beckers et al. [37] analyzed motor learning by examining the individual task performance improvement after training with partners connected by soft and hard springs.

Che et al. [28], Takagi et al. [15], and Beckers et al. [37] found that as the interaction stiffness increases, dyadic task performance improvement (with respect to the solo trial) of the less skilled partner increases. Ganesh et al. [2] reported that the largest task performance improvement occurs when the spring has an intermediate value of stiffness, neither too small nor too large (The stiffness values are given in Additional file 2: Table S3.). All four studies reported that there was no significant performance improvement for the better partner in any of the tested interactive stiffnesses.

Piovesan et al. [29] analyzed the effect of interaction stiffness in a rehabilitation setting where users try to assess the spasticity of simulated hypertonic arms while subjects are virtually connected to them with virtual springs through a single interaction port. It was concluded that a soft connection leads to significantly worse assessment performance, especially when the severity of spasticity is mild.

The effect of the viscosity and damping ratio on dyadic task performance was investigated by Tanaka et al. on a “balance seesaw” task [16]. Participants operate two handles of a robotic device to control the angle of a virtual bar to drop a virtual ball (that rolls on top of the bar) into a moving box below. The virtual mass of the bar was virtually connected to ground through a damper, and the participants’ handles were connected to each side of the bar through a spring and damper. They found that a high damping ratio of the connection to the bar and low viscosity of the handle results in significantly worse task performance. The stiffness and damping values used in each study can be found in Additional file 2: Table S3.

#### Non-physical interaction affects the physical communication

Wang et al. [17] and Takagi et al. [38] investigated the effects of additional non-physical interaction in addition to physical interaction for reaching and force reproduction tasks, respectively. In the study of Wang et al. [17], subjects tried to reach an invisible target while getting directives from their supervisor—who could see the target—with only physical interaction or physical and auditory interaction together. Physical interaction was implemented by mirroring the position of the supervisor’s haptic device to the other subject’s haptic device. Similarly, in the study of Takagi et al. [38], subjects tried to reproduce the force they felt from their partner either while looking at each other (physical and visual interaction) or not looking at each other (physical interaction). Both studies found that task performance with the additional non-physical interaction component was significantly different from the performance with physical interaction alone. While additional visual interaction caused the users to perform worse [38], additional auditory interaction was found to improve performance [17].

#### Increasing the number of non-physical interaction channels increases motivation

Johnson et al. [18] and Le et al. [19] compared the motivation and engagement of subjects for varying numbers of non-physical interaction channels (e.g., no interaction, audio, audio-visual). In the study of Johnson et al. [18], pairs played a competitive tic-tac-toe game with each other while they were connected to an end-effector type robot in one of the three conditions: (1) no interaction with the opponent, (2) subjects could hear each other, and (3) subjects could see and hear each other. Similarly, the subjects in the study of Le et al. [19] collaboratively stacked virtual cubes by using haptic devices while either having only visual feedback or visual and auditory feedback from their partners. Both studies found that the subjects’ interest and motivation increased as the number of non-physical communication channels increased. Moreover, Le et al. [19] reported that when subjects were allowed to talk with each other, they completed their task faster.

### Effects of interaction mode

As defined earlier (see Fig. 1), we consider the following interaction modes: collaborative, cooperative, co-active, and competitive [30, 31].

Most studies investigated the effect of interaction modes on either (1) task performance and individual motor learning or (2) game experience (i.e., motivation and engagement levels) during non-physical interaction [3, 5, 9, 18, 22–24, 32, 42–45]. Among them, five studies focused on the effects of collaborative interaction [3, 9, 23, 43, 45], nine studies addressed competitive interactions [3, 5, 18, 22–24, 32, 42, 43], three studies analyzed cooperative interactions [5, 9, 44], and three studies implemented co-active interaction modes [3, 32, 43]. In five studies, interaction modes were directly compared to one another [3, 9, 23, 32, 43] (e.g., competition vs. cooperation).

Nine studies were conducted to investigate the effect of a specific interaction mode during physical interaction compared to solo performance [2, 6–8, 15, 17, 28, 38, 39]. Among the nine studies, eight of them analyzed the effect of collaborative interaction [2, 6–8, 15, 17, 28, 38] and one study analyzed cooperative interaction [39]. Competition with physical interaction was not considered by any of the analysed references. Details of these studies and a summary of their results can be found in Additional file 2: Tables S1 and S4.

#### Interaction modes affect engagement and task performance

The majority of studies that have been reviewed reported that non-physical interaction improves both task performance and gaming experience for rehabilitation games. Most studies showed that game experience (e.g., motivation, enjoyment level, preference) was improved during non-physical interaction compared to performing the task alone for games such as tic-tac-toe [18], balancing virtual objects on a structure [33], air hockey [32], VR-based cooking [9], arm tracking [5] tasks, and Pong game [3, 22, 24].

In addition to the game experience, improved exercise intensity was observed for the dyadic modes in the studies of Gorsic et al. [3, 24], and Thielbar et al. [5]. Moreover, Mace et al. [33] reported improved task performance for collaborative interaction compared to solo for less-skilled subjects.

On the other hand, Gorsic et al. [45] found no differences between solo mode and collaborative mode on the motivation and exercise intensity of sub-acute stroke survivors exercising with a virtual environment. Mace et al. [33] presented decreased task performance for collaborative interaction compared to solo for more skilled subjects.

#### Specific interaction modes are more effective than others in non-physical interaction

Only a small number of studies compare the effect of different interaction modes within the study. Four studies directly compare interaction modes during non-physical interaction [3, 9, 32, 43]. According to Novak et al. [32], for both healthy and post-stroke participants playing air hockey, two distinct groups emerged: those who liked the competitive mode but did not care for the co-active mode, and those who liked the co-active mode but did not enjoy the competitive mode. Also, the group who preferred competitive mode put higher effort while playing competitive as compared to playing in different modes.

Similar results were reported by Gorsic et al. [3] for a Pong game where almost half of the participants preferred competitive mode over collaborative or co-active modes. Moreover, participants who preferred the competitive game played that mode at a higher exercise intensity compared to other modes, as was also reported by Novak et al. [32].

In another study, Pereira et al. [43] reported that in an object-catching VR game collaborative mode promotes more social involvement, which was measured using the Game Experience Questionnaire (GEQ), as compared to competitive and co-active modes. Similarly, subjects in the study of Gorsic et al. [9] preferred collaboration without any defined roles over cooperation with assigned roles in a VR-based cooking game. All studies described in this section emphasize the importance of selecting the preferred interaction mode to achieve high motivation and a positive experience during non-physical interaction.

#### Collaborative physical interaction affects task performance and individual motor learning

Improved task performance or individual motor learning during physical interaction for collaborative tasks was reported in several studies [2, 4, 7, 8, 15, 17, 41]. These studies were conducted on tracking tasks such as moving a cursor to a dynamic [2, 4, 7, 15] or static [17] target, tracking predefined trajectories [8], or moving a pole back and forth with a specified amplitude [41].

Most studies showed that physical interaction was mutually beneficial; namely, both interacting partners improve task performance during interaction where the task performance in solo trials was compared to the task performance in paired trials. On the other hand, van der Wel et al. [41] found no difference between the task performances of solo and dyadic subjects on a pole moving task. However, it is worth noting that in the solo mode, the other partner was replaced by the other hand of the subject (i.e., bi-manual operation).

Some studies found improved individual motor learning with collaborative haptic interaction compared to training solo under visuo-motor rotation [2] or force field [4]. However, different levels of improvement in individual motor learning were reported depending on the skill level and experience of the partner. More details of the partner characteristics are given in the following section.

### Effects of partner characteristics

Partner characteristics, including the skill level of the partner and the relationship between partners, are also important factors that influence dyadic interaction. Studies have investigated the effects of different skill levels between interacting dyads in collaborative reaching tasks [6, 28] and tracking tasks [2, 7, 8, 12, 37] with physical interaction, as well as the effects of the partner relationship in collaborative or competitive rehabilitation games [3, 22, 33] with non-physical interaction. Details of these studies and the summary of their results can be found in Additional file 2: Table S5.

#### Skill level of the partner affects individual motor learning

Beckers et al. [37], Mireles et al. [6], and Kager et al. [8] investigated the effects of partner’s initial skill level on individual motor learning for planar tracking, reaching, and trajectory following tasks, respectively. All three studies implemented collaborative interaction with a virtual spring between subjects’ upper limbs by robotic manipulanda. While Beckers et al. [37] and Kager et al. [8] implemented visuo-motor disturbances, Mireles et al. [6] implemented a force field that subjects need to adapt to. Beckers et al. [37] found that subjects who trained with more skilled partners obtained better individual motor learning compared to subjects who trained with less skilled partners. On the other hand, Mireles et al. [6], and Kager et al. [8] reported significant results and qualitative trends respectively, suggesting training with a partner with a similar skill level resulted in better individual motor learning compared to training with an expert.

#### Interacting with a better partner improves task performance

Nine studies analyzed the effect of the partner’s skill level on task performance [2, 6–8, 12, 15, 28, 33, 37]. While eight of them implemented collaborative physical interaction between the subjects during tracking [2, 7, 12, 15, 37], reaching [6, 28], and trajectory following tasks [8], one of them only used visual interaction with no physical connection between the subjects during a collaborative balancing task [33]. All of these studies reported that interacting with a better (i.e., more skilled) partner resulted in better task performance during dyadic interaction compared to interacting with a worse partner or performing the same task solo. Some of these studies also found that even interacting with a worse partner improves the task performance during interaction compared to solo performances [2, 7, 12].

#### Interacting with relatives/friends at home raises motivation

Gorsic et al. studied the effects of partnership on motivation and intensity [3] in rehabilitation games, where participants played four variants (competitive, collaborative, co-active, and single-player) of a Pong game with either their friends/relatives at home or with their occupational therapists at a clinic. In another study, Gorsic et al. asked patients to play a competitive Pong game in two different conditions: (1) with a relative/friend at home and (2) with another patient (stranger) at a rehabilitation clinic [22]. Compared to patients exercising with a stranger at a clinic (a therapist or another patient), patients exercising with a friend/relative at home preferred competitive game modes and showed higher motivation and engagement [3, 22].

## Discussion

### Devices and experimental setups

All studies included in this review were based on upper-limb movements or rehabilitation tasks. Most studies were also performed with hand-held end-effector-type manipulators, haptic devices, or sensors where there is a single interaction point between the device and the user. These studies could be extended to lower-limb movements (e.g., tracking trajectories with the ankle for rehabilitation [48]) to investigate if current results can be generalized. Furthermore, conducting studies with multiple interaction points between the device and the user, such as with a multiple DOF exoskeleton that can provide virtual connections of varying and controllable impedance at the joint level between dyads, would aid our understanding of how dyadic interaction affects task performance and motor learning at the joint level.

### Haptic rendering

In most studies on HRH physical interaction, the haptic connections between dyads are virtual spring-damper elements. Other physical interaction schemes, such as magnet-like force fields [21], could be investigated to provide a holistic view of the effects of physical interaction. Lessons can also be learned from the vast amount of previous work in teleoperation [49–52]. A promising possible approach, due to its similarity to traditional rehabilitation, could be to mirror the motion of user A to user B, while only sending the force measurement of user B to user A [53–56]. This type of asymmetric physical interaction can be beneficial, especially in cooperative scenarios where users have different roles. Designing the characteristics of the physical interaction based on the assigned roles could improve the outcomes of the interaction. For example, in a rehabilitation setting, the therapist can follow the patient’s motion through their own device while applying forces as needed to assist or correct the patient instead of just being connected with virtual springs [14].

#### Towards haptic rendering in a network of subjects

While most studies focus on interaction between only two subjects, scenarios where a network of people interact with each other can be quite beneficial, especially in a rehabilitation setting. For example, the ability to simultaneously interact with a network of patients can allow therapists to provide more efficient and effective therapy. Even though it might be expected that adaptation and learning will take more time with more people, especially if the interaction is only non-physical, Takagi et al. [12] obtained promising results related to the benefits of increased group size with physical interaction. They examined the task performance of dyads, triads and tetrads whose hands are connected with virtual springs while they follow a common moving target. They found that individuals use the interaction force to estimate the collective’s target and improve their movement planning. As the group size increases, the variance of haptic inference decreases, which results in better estimate and task performance.

Considering the initial positive results and its possible impact on rehabilitation practice, we believe that it will be beneficial to the field to allow a network of people to interact with each other through their own robotic devices in future studies. In addition to solving the practical challenges of developing these systems, the effect of multilateral HRH interactions on task performance and motor learning should be explored.

### Metrics

Task-related measurements (e.g., completion time, tracking error) are generally used to quantify short-term motor learning due to dyadic training. Motor learning also involves neuroplastic changes in the brain, and it has two key attributes: retention and transfer. These are not studied in the papers we reviewed. With advanced technologies in electroencephalography (EEG), we could potentially measure the neuroplastic changes in the brain during different dyadic interaction conditions and further understand the mechanism of dyadic interactions in motor learning [57, 58]. In addition, it would be useful to include experiments that investigate how the key factors (e.g., interaction type, interaction mode, partner characteristics) in dyadic interactions affect the retention/persistence and transfer/generalization of motor learning, which is essential for rehabilitation purposes.

Two papers [3, 22] examine how the relationship between partners affects their motivation and preferences, and many studies show that motivation can directly influence training intensity, which improves functional outcomes. However, there has been little study of the direct effect of different interaction modes on clinical outcomes (e.g., via the Fugl-Meyer Assessment). Clinical measurements should be used in combination with subjective and objective measurements to assess how key factors in dyadic interaction affect rehabilitation outcomes.

### Effects of the interaction type and characteristics

Twelve studies [2, 15–19, 28, 29, 36–38, 40] that analyzed the effects of interaction type and characteristics mostly reported similar results on dyadic interaction. However, there are several contradicting results and areas that suggest further investigation.

#### Limited studies about the effects of interaction characteristics on individual motor learning

Some tasks can only be performed by two people, so only dyadic task performance can be analyzed. In a rehabilitation setting, however, the goal is motor learning for the individual patient. In other words, in rehabilitation settings, dyadic interaction is used to train people to improve their solo performance. However, most studies investigate the effects of interaction types and characteristics focused on dyadic task performance but do not investigate motor learning of individuals due to dyadic training.

Only Beckers et al. [37] investigated the effect of interaction characteristics (i.e., spring stiffness) on individual motor learning and found no significant learning difference between training with compliant and stiff interaction. Considering the fact that they also did not find any learning difference between solo and stiff connection, it would be valuable to replicate a similar experimental procedure with much stiffer virtual springs or with rigid interaction. This would allow to compare the two extremes of the physical interaction characteristics (no interaction and rigid interaction).

#### Conflicting results on the effect of interaction stiffness

Four studies implemented collaborative target tracking tasks where solo and dyadic tracking performances are compared [2, 15, 28, 37]. While Ganesh et al. [2] found that the biggest dyadic task performance improvement (with respect to solo) occurs at intermediate levels of stiffness between members of a dyad, Che et al. [28] , Takagi et al. [15] and Beckers et al. [37] observed that as the stiffness increases performance improvement increases. (The stiffness values are given in Additional file 2: Table S3.) The reasons for these different results are unclear, but two main differences between the experimental setups might be the source of the conflicting results. First, the tracking tasks in the study of Takagi et al. [15] and Che et al. [28] were 1 DoF, but in the study of Ganesh et al. [2] and Beckers et al. [37], subjects tracked targets that moved in a plane. Second, while in the articles of Ganesh et al. [2], Takagi et al. [15], and Beckers et al. [37] subjects tracked a moving target, in the study of Che et al. [28] subjects reached to a static target. Therefore, additional studies with different task spaces and target types (moving, static) could be of help to better understand the effect of interaction stiffness on task performance.

#### Little study of the effect of interaction damping

Even though several studies [2, 28, 29, 37] implemented virtual dampers between subjects for stability, they were focused on the effect of different spring stiffnesses on dyadic task performance. Only Tanaka et al. [16] analyzed the effects of virtual damping by varying its viscosity. They found significantly different task performance at different viscosities and damping ratios. Additional study of the effect of velocity-dependent forces on task performance is warranted.

Several studies have reported promising motor learning results for single-person upper-limb reaching or tracking tasks due to training under destabilizing negative viscosity [59–61]. Negative viscosity can improve motor learning by increasing motor variability and facilitating the development of internal models of body dynamics [62, 63]. However, none of the references reviewed in this paper implemented negative viscosity between the subjects.

### Effects of the interaction mode

#### Many factors contribute during non-physical interaction

It is clear that, when the interaction mode is selected properly, non-physical interaction can improve the participants’ motivation, engagement, and gaming experience, leading to an increase in physical activity and training intensity. Many of the included studies confirmed that the personality of the user, the intimacy between dyads, and environmental factors play important roles in choosing the proper interaction mode. In neuromuscular therapy, training intensity—alongside early treatment and user-centered, task-oriented training—is a key factor for functional improvement. Therefore, non-physical interaction has great potential to further increase the benefits brought by robot-assisted neuromuscular and virtual reality-assisted therapy.

#### Comparison of interaction modes during physical interaction is needed

Despite the increasing interest in comparing different interaction modes for non-physical interaction conditions, no study compares how different physical interaction modes affect task performance or individual motor learning. Moreover, no study has implemented competitive physical interaction between subjects among the selected studies.

Even though competitive physical interaction would result in worse task performance during the training, it is quite promising for improved individual motor learning. Considering the positive results obtained by haptic error modulation and resistive training studies [64–67], competitive interaction can facilitate error-based motor learning. One possible way to implement competitive physical interaction is rendering virtual springs with negative stiffness between the subjects such that they push each other away while following the same target. It is also possible to facilitate competitive physical interaction by assigning conflicting goals while subjects haptically interact with each other [48, 68].

Cooperative interaction in a teacher-student scenario can also be helpful for individual motor learning, especially for less-skilled subjects who are not able to complete the task alone. One way to obtain this interaction is by implementing a virtual uni-directional spring where force is transmitted only to the student. For a perfect teacher, this interaction resembles assistive robotic training strategies [69–71].

Due to their relevance to different robot-aided physical therapy strategies, implementation of competitive, collaborative, and cooperative physical interaction in a single study to compare the effects on individual motor learning could be very beneficial to our understanding of HRH interaction. We believe that such results could be further translated to robotic controllers that can mimic human-like adaptability.

### Effects of partner characteristics

The studies analyzed here were focused on either effects of partner skill level on task performance/individual motor learning or effects of partner relationship on exercise intensity and motivation for rehabilitation scenarios.

#### Limited and conflicting results about the effects of partner characteristics on individual motor learning

Only three of the included studies investigated the effects of the training partner’s initial skill level on individual motor learning [6, 8, 37]. All of them implemented collaborative physical interaction between the subjects during planar tracking/reaching tasks under visuo-motor disturbances [8, 37] or force field [6].

Kager et al. [8] and Mireles et al. [6] reported qualitative trends and statistically significant results respectively, suggesting training with a partner that has a similar initial skill level improves individual motor learning more compared to training with an expert. The reason behind these results might be that novice partner does not need to work hard when they are connected to an expert, which can negatively impact their learning of the task. On the other hand, Beckers et al. [37] found that as the initial skill level of the training partner increases, individual motor learning increases. While the hypothesis behind this result might be that having a better teacher is better for learning new tasks, it is worth noting that it was a collaborative task with no explicit roles (e.g., teacher-student) given to the subjects. Another possible explanation is the fact that initially less-skilled partners, who are more likely to interact with better partners, have more room to improve their individual performance than initially more skilled partners. Another important difference between these studies is that Beckers et al. [37] use the same trajectory for the dyadic and solo trials with only different starting points. Therefore, cognitive memory might play a role in their results. On the other hand, Kager et al. [8] and Mireles et al. [6] use different trajectories or random targets where results are more generalizable to different motions and depend less on the properties of the selected trajectory. Moreover, while Beckers et al. [37] measured motor adaptation, Mireles et al. [6] and Kager et al. [8] investigated mostly skill learning. We believe more work on this topic is warranted with different tasks, different disturbances, and more variety on the initial skill differences of the subjects to better understand the reasons behind the conflicting results.

### Transferring results to rehabilitation environments

#### Long-term effects need further investigation

Physical rehabilitation is usually a process that takes weeks, months, or even years. Among selected studies, only five of them implemented experimental setups where training takes place in multiple sessions during different days or weeks [5, 6, 22, 23, 45]. Moreover, only Mireles et al. [6] implemented physical interaction and had a post-retention session on a different day from the last training session. Extending the number of studies involving physical dyadic training over a long period of time and investigating long-term retention could inform the application of results to rehabilitation scenarios.

#### Independent investigation of partner’s nature and environmental settings is missing

In rehabilitation settings, in addition to the partner’s nature, environmental settings can influence performance and motor learning. In the studies of Gorsic et al. [3, 22], more positive results in terms of motivation and exercise intensity were obtained for participants interacting with a friend/relative at home compared to interacting with a stranger (another patient or therapist) at a clinic. However, it is worth noting that whether the increased motivation was due to interacting with a relative/friend or due to the environmental factors was not analyzed. Additional studies that independently investigate the effects of the partner’s nature and environmental settings are needed. Results from these studies can be translated to possible home or clinic-based dyadic rehabilitation.

#### Lack of studies on the effects of partner’s age and impairment

While there are studies analyzing different kinds of tasks, skill levels, and interaction environments, no study examined subjects’ ages among the selected references. We believe this factor should also be investigated in future studies considering that robotic haptic guidance results in different motor learning improvements among different ages [72, 73]. Another factor that might influence preference and motivation is the participants’ level of impairment. Clinical trials need to include more participants with different levels of impairment for a systematic investigation. Lessons learned regarding these factors can be applied in clinical scenarios where patients interact with each other during their training sessions.

### Limited variation of interaction condition

Even though the effects of interaction type, interaction mode, and partner characteristics are investigated in different studies, only a couple of studies examine more than one of these three components with the same experimental devices and procedure. Moreover, as shown in Fig. 3, there is no single study that systematically varies each interaction component. To have more generalizable results on task performance and individual motor learning, we believe that interaction types, characteristics, and modes should be systematically changed, and the effects of a wide variety of interaction conditions should be compared.

### Does physical HH interaction improve task performance?

Many studies clearly conclude that collaboratively and physically interacting with a better partner results in better task performance than interacting with a worse partner for different tracking, reaching, or trajectory following tasks at upper limb with different physical interaction characteristics (i.e., stiffness, damping). This is not surprising due to the nature of collaborative physical interaction where the partner’s individual performance directly affects the dyadic task performance.

Interestingly, some studies found that even connecting with a worse partner can improve task performance [2, 7]. Subjects using haptic forces to estimate their partner’s target to improve their own prediction of the target can be one explanation for that [11]. It is also possible that physical interaction corrects the “*irregular or erratic tracking behaviours*” of subjects [7]. Implicit role specialization [1] between subjects might be another explanation for the improved task performance compared to solo even if the partner’s skill level is worse.

### Does physical HH interaction improve individual motor learning?

Unlike task performance, the results obtained on the effects of HH interaction on individual motor learning are more limited, contradictory, and hard to generalize. Three studies [2, 7, 37] that compared collaborative dyadic training with solo training did not report consistent results with each other. Ganesh et al. [2] and Beckers et al. [37] implemented an almost identical experimental protocol for a target tracking task with visuo-motor rotations. While Ganesh et al. [2] reported better individual motor learning with dyadic training than solo training, Beckers et al. [37] did not find any significant difference between the individual motor learning of the dyadic group or solo group.

One possible explanation for the different results might be the fact that different robotic devices were used. Neither article reported their haptic transparency or virtual environment rendering fidelity during solo and connected trials, respectively. It is important that interaction forces are rendered with high accuracy to correctly compare the results from different interaction conditions. For example, if parasitic forces felt by the user in solo mode (e.g., frictional and inertial forces) are significant compared to the interaction forces in the connected mode, the comparison of solo and connected training can be misinterpreted. Similarly, if the stiffness felt by the users is not consistent during dynamic motion, the effects of the physical interaction might be misinterpreted.

Due to the conflicting and limited number of studies, we believe further systematic studies focusing on individual motor learning are warranted. Moreover, presenting haptic transparency, and rendering performances can be beneficial to prove solo and dyadic conditions are implemented properly.

### Human-like robotic controllers

Further analysis of how humans move when they are coupled to others, and transferring this knowledge to human-robot systems, can lead to advanced human-like robotic controllers. Human-like robot controllers based on improving motor learning could have significant implications on rehabilitation robotics to achieve or perhaps improve the results obtained by conventional human therapists. Moreover, knowledge of HH interaction on dyadic task performance can be used to develop robots that work together with humans in industry settings, such as carrying a large object or performing assembly tasks together.

We found several studies that did not meet our inclusion criteria, but which did investigate HH interaction to develop human-like robotic controllers or to understand dyadic communication. One way to represent human motion is to model it as an optimal controller minimizing a cost function that penalizes error and effort [11, 68, 74, 75]. Another less commonly used method is to represent the human as a first- or second-order system with delay [76, 77]. It is also important to take specialization, role sharing, and negotiation into account while modeling human behavior. Accelerator/decelerator [1], executor/conductor [78], and leader/follower [68] are some of the observed roles in human-human studies. In line with these results, Kucukyilmaz et al. implemented a haptic role exchange mechanism on a dynamic human-robot joint manipulation task and showed that the role exchange mechanism improves task performance and joint efficiency of the partners [79, 80].

Three studies used HH interaction-derived robot controllers to substitute one of the peers [11, 81, 82]. In all of these studies the subjects either did not realize that they were interacting with a robot [81, 82] or their task performance or individual motor learning results with robot partners were similar to their results with human partners [11]. However, it is worth noting that it was not clear if substituting one of the peers had an effect on the motivation because the experimental procedures did not involve any social interaction component. We believe that these results are promising for obtaining human-like robotic controllers, and they should be supported with additional studies with different interaction conditions and tasks.

## Conclusions

Many variables govern HH interaction, including interaction type (i.e., physical, non-physical or the combination), characteristics (i.e., interaction impedance between the individuals), mode (i.e., collaborative, cooperative, co-active, competitive), and partner characteristics (i.e., skill level, intimacy, personality). Over the past few decades, many researchers have been systematically investigating each variable to better understand why humans physically interact so effectively and to improve individual motor learning in HRH systems.

Studies on interaction type showed that additional physical interaction resulted in better task performance than visual interaction alone during tracking tasks. Some studies reported that the type of non-physical interaction (e.g., visual vs. auditory interaction) can greatly influence task performance. The combination of additional non-physical communication channels (e.g., visual vs. visual and audio interaction) with physical interaction was also shown to influence the motivation level during a given task. Some studies also emphasized the significance of the characteristics of the physical interaction (e.g., interaction stiffness).

Similarly, the choice of interaction mode was also shown to have significant effects on task performance and engagement. Interaction modes were mostly studied during non-physical interaction, showing that despite the mode, all types of non-physical interaction can improve engagement and motivation when compared to performing the same task alone. Depending on the personality of the individual, competitive or cooperative modes led to enhanced motivation and movement intensity. Partner characteristics also played a prominent role in task performance and motor learning. Interacting with relatives and friends increases motivation when compared to interacting with a stranger. There were also several studies showing that physically interacting with a more skilled partner in a collaborative arm tracking task improves the task performance while interacting with a partner that has a similar skill level improves individual motor learning.

Although it is difficult to draw clear conclusions as to which interaction type, mode, or partner characteristic may lead to optimal task performance or individual motor learning, these results show the possibility for improved motor learning during HH or HMH interaction. By selecting the optimal combination and personalizing these variables, there is a clear opportunity to not only enhance motor learning during HH or HMH interactions but to also make the experience more enjoyable. Future studies could explore human interaction with a multi-joint robot at multiple contact points (e.g., an exoskeleton) in order to analyze the motor task at the joint level. With the continuous advances in neuroimaging tools, such as EEG, it may be possible to monitor changes in the brain with high spatial and temporal resolution. Investigating the effects of interaction type, mode, and partner characteristics at the cortical level may provide insight into the mechanism of motor improvement during physical and non-physical interaction.

## Supplementary Information

**Additional file 1.** Search query.

**Additional file 2.** Experimental details and summaries.

## Data Availability

Not applicable.

## Abbreviations

HH: Human-human
HRRH: Human-robot-robot-human
HRH: Human-robot-human
HMH: Hman-machine-human
