# Supplementary material for: Human-machine-human interaction in motor control and rehabilitation: a review
Source: J Neuroeng Rehabil. 2021 Dec 27;18:183. doi: 10.1186/s12984-021-00974-5 (PMC8714449; doi:10.1186/s12984-021-00974-5)
Supplement: Supplementary file 1 — Additional file 1. Search query. [file 12984_2021_974_MOESM1_ESM.docx]

**Additional File 1 – Search Queries used in the systematic search**

| **Database** | **Search Query** |
| --- | --- |
| **Scopus** | ALL ( ( exoskeleton  OR  robot  OR  robotics ) )  AND  ALL ( ( "haptic interaction"  OR  "physical interaction"  OR  "social interaction"  OR  motivation  OR  game ) )  AND  ALL ( ( rehabilitation  OR  therapy  OR  "motor control"  OR  "motor learning" ) )  AND  TITLE-ABS-KEY ( ( pairs  OR  dyads  OR  multiplayer  OR  telerehabilitation  OR  tele-rehabilitation ) ) |
| **IEEE Xplore** | ((("Full Text & Metadata":exoskeleton OR "Full Text & Metadata":robot OR "Full Text & Metadata":robotics ) AND ("Full Text & Metadata":"haptic interaction" OR "Full Text & Metadata": "physical interaction" OR "Full Text & Metadata":"social interaction" OR "Full Text & Metadata": "motivation" OR "Full Text & Metadata": "game") AND ("Full Text & Metadata":rehabilitation OR "Full Text & Metadata": therapy OR "Full Text & Metadata": "motor control" OR "Full Text & Metadata": "motor learning" ) AND ("All Metadata":pairs OR "All Metadata":dyads OR "All Metadata":multiplayer OR "All Metadata":telerehabilitation OR "All Metadata": tele-rehabilitation))) |
| **PubMed** | ((((exoskeleton OR robot OR robotics) ) AND ("haptic interaction" OR "physical interaction" OR "social interaction" OR motivation OR game)) AND (( rehabilitation OR therapy OR "motor control" OR "motor learning" ))) AND (( pairs OR dyads OR multiplayer OR telerehabilitation OR tele-rehabilitation )) |
