# Supplementary material for: Human-machine-human interaction in motor control and rehabilitation: a review
Source: J Neuroeng Rehabil. 2021 Dec 27;18:183. doi: 10.1186/s12984-021-00974-5 (PMC8714449; doi:10.1186/s12984-021-00974-5)
Supplement: Supplementary file 2 — Additional file 2. Experimental details and summaries. [file 12984_2021_974_MOESM2_ESM.pdf]

Additional File 2 - Experimental details and summaries of the selected references

Table 1 Characteristics of the papers included in the qualitative analysis. Subcategories are based on their research question. What is the effect of (1) interaction type/characteristics, (2) interaction mode, and (3) partner's characteristics

Reference	Task	Device	Metrics	Subcategories
Wang et al., 2016 [17]	Trying to reach an invisible target via controlling a mouse by dominant hand while getting directives orally or through a haptic device.	Phantom Omni and mouse	• Trajectory of the mouse and haptic devices<br>• Time to complete the task<br>• Path length, overshoot, RMS error<br>• Verbalization	1
Takagi et al., 2016 [38]	Exchange force with their partners. The goal is to reproduce the force they feel as accurately as possible.	Hi5 robotic lever	• Exchanged and reproduced force	1
Le et al., 2016 [19]	Controlling the pose of the two sticks connected to the two edges of a cube. Purpose is to stack the cubes on top of each other.	Phantom Omni haptic devices	• Interest/enjoyment, perceived competence, dis/satisfaction by IMI<br>• Hand position and interaction force<br>• Time to complete the task<br>• Stress indicator measured by Galvanic skin sensors	1
Chellali et al., 2010 [40]	Learning a biopsy task. Training takes place by interacting with instructors in 3 different ways. Then, subjects conduct the biopsy task with different partners; and performances from 3 different learning groups are compared.	Virtuose 6D desktop haptic arms	• Conversations<br>• Planning strategy<br>• Needle insertation time<br>• Number of contact with the organs	1
Liu et al., 2013 [36]	Controlling a virtual sphere inside a curvy pipe as dyads. Each subject can only control the motion of the sphere along one axis. Their purpose is to move the sphere as fast as possible with the least amount of collision.	Omega and Phantom haptic devices	• Time to complete the task<br>• Collision with the pipe<br>• Participant's feeling of copresence obtained by a questionnaire	1
Takagi et al., 2018 [15]	Tracking a moving target on an arc. Subjects experience both solo and connected trials. During connected trials, they follow the same target with their own separate cursor, while their wrists are virtually connected.	Hi5 robotic lever	• RMS tracking error<br>• Performance improvement as the change in tracking error between solo trial and connected trial<br>• EMG at FCR and ECRL muscles	1 , 3
Tanaka et al., 2019 [16]	Controlling the angle of a bar to drop a ball from the top of the bar into a laterally moving box. Subjects complete this task as dyads while haptically connected.	A robotic system with a pair of handles composed of force-torque sensors and linear motor tables	• Success ratio<br>• Subjective preference of different assistive strategies	1
Piovesan et al., 2013 [29]	Assessing the severity of a simulated hypertonic arm that is virtually connected to subject's hand.	2 DoF robotic manipulandum	• Correctness of the assessment	1
Johnson et al., 2008 [18]	Playing tic-tac-toe	Gentle and ADLER rehabilitation devices	• Interest/enjoyment by IMI<br>• User preference<br>• Position,velocity, and force at the wrist	1, 2
Ganesh et al., 2014 [2]	Tracking a moving target on a monitor under visuo-motor rotation. Subjects experience both solo and connected trials. During connected trials, they follow the same target with their own separate cursor, while their manipulanda are virtually connected.	A dual robotic system moving on the plane holded by the end-effector	• Mean tracking error<br>• Performance improvement as the change in tracking error between solo trial and connected trial	1, 3
Che et al., 2016 [28]	Moving indicator to the center of the static target as fast as possible. Subjects complete this task both solo and as dyads while haptically connected. Subjects follow the same target while only seeing their own indicator.	da Vinci Research Kit	• Completion time<br>• Performance improvement: time difference between individual condition and colloborative condition<br>• Interaction Force	1, 3
Beckers et al., 2020 [37]	Tracking a moving target on a monitor under visuo-motor rotation. Subjects experience both solo and connected trials. During connected trials, they follow the same target with their own separate cursor, while their manipulanda are virtually connected.	A dual robotic system moving on the plane holded by the end-effector	• Mean tracking error<br>• Performance improvement as the change in tracking error between solo trial and connected trial	1, 3

Table 1 Characteristics of the papers included in the qualitative analysis. Subcategories are based on their research question. What is the effect of (1) interaction type/characteristics, (2) interaction mode, and (3) partner's characteristics (*continued*)

Reference	Task	Device	Metrics	Subcategories
Gorsic et al., 2018 [9]	Cooperative cooking game in VR environment with wrist and forearm movement (move hand module forward, backward, left or right by 20 degrees). Subjects performed game in single-player or cooperative mode. In cooperative mode, subjects' roles are either fixed or undefined.	Bimeo arm and a commercially available joystick	• Subject experience (IMI)<br>• Exercise intensity	2
Thielbar et al., 2020 [5]	A Virtual Environment for Rehabilitative Gaming Exercise (VERGE) system, consisted of three different arm tracking games was tested. Subjects either played in single-user mode or multi-user mode.	Kinect	• Subject experience (IMI)<br>• Arm displacement<br>• Time spent<br>• Fugl-Meyer Assessment of Motor Recovery After Stroke Upper Extremity	2
Novak et al., 2014 [32]	Air hockey game with shoulder abduction and adduction. Subjects either played in single-player, competitive, or cooperative mode.	ARMin III	• Subject experience (IMI)<br>• Subject personality (IPIP and Revised Competitiveness Index)	2
Gorsic et al., 2019 [23]	Pong game and cooking game with wrist and forearm motion (tilting hand module forward and backward and left and right by 20 degrees). Subjects either played in competitive or cooperative mode.	Bimeo arm	• Subject experience (IMI)<br>• Game choice, duration, maximum difficulty Competitiveness Index<br>• Exercise intensity	2
Gorsic et al., 2020 [24]	Pong game with wrist and forearm movement (move hand module left and right by 20 degrees). Subjects played either with a simple computer opponent, "human-like" computer component, human opponent, and a simple computer opponent accompanied by a human to chat with.	Bimeo arm and a commercially available joystick	• Subject experience (IMI)<br>• Exercise intensity	2
Oosterhout et al., 2018 [39]	(1) Maneuvering an object through a bounded path and (2) mounting an object in VR environment. Subjects either performed the task in dyads (collaboration), using bi-manual control or using uni-manual control.	Haption Virtuoso 6D and a comsumer-graded USB joystick	• Completion time<br>• Shortest distance to contact<br>• Spectral arc length<br>• Trajectory length	2
Ventura et al., 2019 [44]	Classify objects by labeling and trashing irrelevant labels in a Web-based citizen science platform. The labeling and trashing roles were divided by two subjects so that the two can cooperate.	Novint Falcon	• Number of labels processed<br>• Time spent<br>• Movement speed<br>• Total path length<br>• Enjoyment level survey	2
Gorsic et al., 2017 [42]	Two-player competitive Pong game. A ball bounces across the screen, and players have to intercept it with their paddle by moving left and right so that the ball does not reach the top or bottom of the screen.	Bimeo arm and a commercially available joystick	• Intrinsic Motivation Inventory (IMI)<br>• Hand velocity	2
Pereira et al., 2019 [43]	Catching balls that appeared at random positions in a VR environment. Dyads played in either competitive, co-active or collaborative gaming modes.	Playstation Eye Camera and two handles	• Game experience questionary (GEQ)<br>• International Personality Item Pool (IPIP)<br>• Montreal Cognitive Assessment	2
Batson et al., 2020 [4]	2D tracking task. Subjects experience both solo and haptically connected trials under the presence and absence of a simple force field.	Geomagic Touch haptic devices	• Mean tracking error	2
Gorsic et al., 2020 [45]	Moving objects to designated spots for preparing meals and sorting dishes in a virtual environment	Bimeo arm	• Exercise intensity<br>• Motivation	2
van der Wel et al., 2011 [41]	Moving a pole back and forth between two targets at different amplitudes and frequencies. Participants completed the task either bimanually or as dyads.	Rigid pole with two cables on the sides allowing 1 DoF motion.	• Mean absolute end point error	2

Table 1 Characteristics of the papers included in the qualitative analysis. Subcategories are based on their research question. What is the effect of (1) interaction type/characteristics, (2) interaction mode, and (3) partner's characteristics (*continued*)

Reference	Task	Device	Metrics	Subcategories
Gorsic et al., 2017 [3]	Playing variants of classic game of Pong for arm rehabilitation. Each participant played four games (1 competitive, 2 co-operative (shared field and split field), and 1 single-player game) with either relative/friends or therapists.	Bimeo arm and a commercially available joystick	• Intrinsic Motivation Inventory (IMI)<br>• Exercise intensity	2, 3
Gorsic et al., 2017 [22]	Pong game with wrist and forearm movement (move hand module left and right by 20 degrees). Subjects played either with an unimpaired friend or relative at home or with other subjects at a rehabilitation clinic. Single-player or competitive multiplayer mode was played.	Bimeo arm and a commercially available joystick	• Subject experience (IMI)<br>• Overall subject experience questionnarie<br>• Conversational level<br>• Game choise, duration, maximum difficulty<br>• Exercise intensity	2, 3
Mace et al., 2017 [33]	Balancing a dynamic ball on a beam to collect 'points' by controlling each end of the beam using independent digital force-sensing handgrips. Healthy subjects were paired with either healthy subjects or people with hemiparetic stroke.	Force-sensing handgrips	• Points in game<br>• Error to the target<br>• RMSE of forces<br>• Smoothness as the derivative of the filtered force<br>• Intrinsic Motivation Inventory (IMI)	3
Mireles et al., 2017 [6]	Reaching a target position by controlling the tip of a virtual tool under position dependent force-field. Subjects performed the task under five conditions: naive-naive group with/without bi-manual prior, naive-expert group with/without bi-manual prior, and bi-manual control group.	"Braccio di Ferro" robot	• Effort index (force exertion)<br>• Completion time<br>• Mutual information<br>• Average RMS of EMGs	3
Kager et al., 2019 [8]	Tracking a figure-eight path with visuomotor rotation of 80 degrees. Subjects performed the task either virtually connected with novice/expert partner or without virtual connection.	Planer upper limb robot 'H-Man'	• Completion time<br>• Tracking error	3
Takagi et al., 2019 [12]	Tracking a moving target on the screen with varying trajectories. Subjects were asked to track the target as accurate as possible under 4 conditions (solo, dyad, triad, tetrad) with different skill-level partners.	Phantom 1.5HF	• Tracking error<br>• Performance improvement	3
Beckers et al., 2018 [7]	Tracking randomly appearing targets with arm movements in a 2D plane over a workspace that was constricted to a circle with a radius of 10 cm. Subjects performed the tracking task either solo or haptically connected in dyads.	2 DoF robotic manipulandum	• Tracking error<br>• Motor learning curve	3

Table 2 Brief summaries of the included references where the effect of the interaction type is investigated. *Results* column does not include all the results of a study but only shows the results related to the effect of different interactions types.

Reference	Interaction type	Results
Johnson et al., 2008 [18]	• No interaction: Subjects only see the game board<br>• Auditory: Subjects can hear each other.<br>• Auditory + Visual: Subjects can hear and see each other.	• Interest and motivation of the subjects are increased as the number of non-physical interaction component increases.<br>• When there is no interaction, subjects move their hand less, in terms of total displacement.
Wang et al., 2016 [17]	• Haptic (H): Supervisor uses a haptic device whose position is mirrored to the subject's device. The subject feels the motion of the supervisor's device by their non-dominant hand.<br>• Verbal (V): Supervisor gives oral directives about the the relative position of the target to the cursor.<br>• Haptic + Verbal (HV): Combination of (H) and (V)	• (VH) and (V) interaction types resulted to significantly better completion time, overshoot and RMS error compared to (H).<br>• (H) caused shortest reaction time to initiate the motion
Takagi et al., 2016 [38]	• Haptic: Subjects do not see each other but only feel the force produced by them through robotic lever<br>• Haptic + Visual: Subjects see each other in front of them and feel the force produced by each other through robotic lever	• Additional visual feedback by seeing the partner strongly influenced the dyads and caused them to under reproduce the force that they feel.
Le et al., 2016 [19]	• Manipulating real objects without talking to each other<br>• Manipulating real objects while talking to each other<br>• Manipulating virtual objects without talking to each other<br>• Manipulating virtual objects while talking to each other	• User engagement and enjoyment was higher while manipulating virtual cubes<br>• Stacking real cubes on top of each other took less time compared to stacking virtual cubes<br>• In virtual case, subjects completed the task faster if they are allowed to talk
Chellali et al., 2010 [40]	• Paper Instruction(PI): Instructor only use verbal instructions and static images<br>• Visual Instruction(VI): Subject see directly the hand of instructor and the feedback on the screen<br>• Visual-haptic Instruction(VH): In addition to VI, subject feels the hand of the instructor through their own haptic device which is virtually connected to the haptic device of the instructor.	• Subjects trained in (VH) group completed the task faster and with less organ contacts(a desirable thing in biopsy) compared to (PI) and (VI) groups<br>• No significant difference has found between the performance of the subjects in (PI) and (VI) groups.
Liu et al., 2013 [36]	• Visual: Subject can see the sphere on the monitor. There is no interaction between the haptic devices of subjects.<br>• Visual + haptic: In addition to the visual feedback from the monitor, users can feel the motion of the haptic device along the axis controlled by their partner.	• With visual-haptic interaction, significantly faster motion with less collision is obtained<br>• On a subjective questionnaire, users indicated that they would prefer visual-haptic interaction

Table 3 Brief summaries of the included references where the effect of the haptic interaction characteristic is investigated. *Results* column does not include all the results of a study but only shows the results related to the effect of different haptic interactions. ζ: damping ratio, c: viscosity, k: spring stiffness

Reference	Rendered haptic interaction	Results
Ganesh et al., 2014 [2]	• $k = 60$ N/m<br>• $k = 120$ N/m<br>• $k = 180$ N/m	• For the worse partner, the biggest performance improvement between solo and dyadic trials is obtained with the medium spring.<br>• None of the haptic interaction characteristics caused any significant performance change for the better individuals compared to their solo performance
Takagi et al., 2018 [15]	• $k = 0.3$ Nm/rad<br>• $k = 1.7$ Nm/rad<br>• $k = 17.2$ Nm/rad	• For the worse partner, the biggest performance improvement between solo and dyadic trials is obtained with the hard spring.<br>• None of the haptic interaction characteristics caused any significant performance change for the better individuals compared to their solo performance<br>• Better partners exerted significantly higher forces compared to their solo performance when the interaction stiffness is medium or high.
Che et al., 2016 [28]	• $k = 40$ N/m<br>• $k = 120$ N/m	• Stiff haptic interaction caused significant performance improvement for the worse individuals compared to their solo performance<br>• Stiff haptic interaction did not cause any significant performance change for the better individuals compared to their solo performance<br>• Weaker haptic interaction did not cause any significant performance change for the better or worse individuals compared to their solo performance
Tanaka et al., 2019 [16]	• $\zeta = 0.5$, $c = 10$ Ns/m<br>• $\zeta = 0.5$, $c = 30$ Ns/m<br>• $\zeta = 1.5$, $c = 10$ Ns/m<br>• $\zeta = 1.5$, $c = 30$ Ns/m	• 3rd haptic environment leads to the least success rate<br>• 1st and 2nd interaction leads to smaller amplitude motion<br>• 1st and 3rd interaction leads to more oscillatory motion
Piovesan et al., 2013 [29]	• $k = 1050$ N/m<br>• $k = 1625$ N/m<br>• $k = 2200$ N/m	• Soft connection leads to a significantly worse assessment of the hypertonic arm if the simulated severity is very mild
Beckers et al., 2020 [37]	• $k = 120$ N/m<br>• $k = 250$ N/m	• There was no significant difference on the individual performance improvements of subjects who trained with soft or hard connection<br>• Stiffer connection resulted in better dyadic performance

Table 4 Brief summaries of the included references where the effect of the interaction mode is investigated. *Results* column does not include all the results of a study but only shows the results related to the effect of different interaction modes.

Reference	Interaction modes	Results
Gorsic et al. 2017 [3]	• Solo<br>• Competitive<br>• Collaborative<br>• Co-active	• The majority of participants with chronic arm impairment preferred either competitive or cooperative games over playing it alone.<br>• Participants who chose the competitive game as their favorite showed increased motivation and exercise intensity compared to other modes.<br>• Participants who chose a non-competitive game as their favorite also showed increased motivation in cooperative games, but not increased exercise intensity.
Gorsic et al. 2018 [9]	• Solo<br>• Cooperative<br>• Collaborative	• Unimpaired participants preferred cooperating over exercising alone and feel less pressured when cooperating.<br>• Participants preferred collaboration without any defined roles over cooperation with defined roles<br>• no significant decrease in exercise intensity was observed when cooperating.
Thielbar et al. 2020 [5]	• Solo<br>• Cooperative<br>• Competitive	• For VR rehabilitation, participants spent significantly more time training in the interactive modes than in the solo mode.<br>• While training with different interactive modes, arm displacement and FMA-UE significantly improved compared to solo mode.
Johnson et al. 2008 [18]	• Solo<br>• Competitive	• A clear positive trend existed in favor of the interactive mode compared to solo mode, which subjects found more valuable, interesting, and enjoyable, and was therefore willing to spend more time at the task.
Gorsic et al. 2017 [22]	• Solo<br>• Competitive	• Participants (with chronic arm impairment) who played against an unimpaired friend or relative at home tended to prefer competition, and exhibited higher enjoyment and exercise intensity when competing than when exercising alone.<br>• Participants (in the acute or subacute phase of stroke) who played against each other in the clinic, however, did not exhibit significant differences between competition and exercising alone.
Novak et al. 2014 [32]	• Solo<br>• Competitive<br>• Co-active	• Nearly all unimpaired subjects preferred playing the interactive modes to solo modes.<br>• There were two distinct player groups: one liked the competitive mode but not the co-active mode while the other liked the co-active but not the competitive mode.<br>• Unimpaired subjects who liked the competitive mode put significantly more effort into it than into the other modes.<br>• Results from impaired subjects were similar.<br>• The subjects' personalities roughly predicted which mode they would prefer.
Gorsic et al. 2019 [23]	• Collaborative<br>• Competitive	• Participants exhibited high motivation and consistent exercise intensity over long-term rehabilitation sessions when performing rehabilitation games in interactive modes.
Batson et al. 2020 [4]	• Solo<br>• Collaborative	• Dyadic group reduced their tracking error with learning both in the absence and presence of the force field while the solo group reduced their tracking error only in the absence of the force field.

Table 4 Brief summaries of the included references where the effect of the interaction mode is investigated. *Results* column does not include all the results of a study but only shows the results related to the effect of different interaction modes. (*continued*)

Reference	Interaction modes	Results
Gorsic et al. 2020 [24]	• Solo (simple computer opponent)<br>• Solo (human-like computer opponent)<br>• Solo (simple computer opponent and another participant to chat with)<br>• Competitive	• The human opponent was the favorite for the majority of participants and resulted in the highest interest/ enjoyment and exercise intensity.<br>• All participants preferred the human opponent over the computer opponent accompanied by a human companion.<br>• Majority of the participants preferred the "human-like" computer opponent over the simple one.
Van Oosterhout et al. 2018 [39]	• Solo (uni-manual)<br>• Solo (bi-manual)<br>• Cooperative	• Two participants who cooperated perform the maneuvering and mounting task faster than either bi- or uni-manual solo mode.<br>• Compared to cooperative mode, uni-manual operators required more control activity for the vertical crane and less for the robotic arm.
Ventura et al. 2019 [44]	• Solo<br>• Cooperative	• Depending on the type of termination, cooperation was not always positively associated with engagement, enjoyment, and motor performance.<br>• Enhancing user engagement, satisfaction, and motor performance through cooperative citizen science tasks relies on both the degree of interdependence among users and the perceived nature of the task.
Gorsic et al. 2017 [42]	• Competitive<br>• Competitive (manual difficulty adaptation)<br>• Competitive (auto difficulty adaptation)	• Both manual and automatic difficulty adaptation lead to higher motivation and exercise intensity than no adaptation.<br>• Unimpaired participants showed no clear preference between manual and automatic adaptation while the majority of impaired participants preferred automatic adaptation
Pereira et al. 2019 [43]	• Collaborative<br>• Competitive<br>• Co-active	• Collaborative game mode elicited significantly higher social involvement in terms of empathy, positive affect, and behavioral involvement compared to competitive or co-active modes.<br>• Collaborative mode seems to be the most adequate choice to be used in multiplayer rehabilitation settings, where social interaction is intended
Gorsic et al. 2020 [45]	• Solo<br>• Collaborative	• 11 of 20 participants preferred collaborative over solo exercise while only 4 preferred solo exercise.<br>• There were no differences in motivation, exercise duration, or exercise intensity between solo and collaborative sessions.
van der Wel et al. 2011 [41]	• Solo (bi-manual)<br>• Collaborative	• There was no significant difference between the task performances of participants who completed the task bi-manual and as dyads.

Table 5 Brief summaries of the included references where the effect of the partner characteristics is investigated. *Results* column does not include all the results of a study but only shows the results related to the effect of different partner characteristics.

Reference	Partner characteristics	Results
Ganesh et al. 2014 [2]	• Novice participant<br>• Expert participant	• Participants connected with an expert have less performance improvement than participants connected with a novice partner.<br>• Compared to solo, dyadic performance improves regardless of the partner characteristics.
Gorsic et al. 2017 [3]	• Patient participant<br>• Patient's friend<br>• Therapist participant	• Patient participants playing with their friends/relatives are more likely to play competitive game than these playing with therapists.<br>• Patient participants playing competitive game gain more exercise intensity these playing cooperative game.
Gorsic et al. 2017 [22]	• Patient participant<br>• Patient's friend<br>• Stranger participant	• Patient participants playing with their friends/relatives show higher enjoyment and exercise intensity than playing alone.<br>• Patient participants have no significant difference in enjoyment or exercise intensity between playing with strangers and playing along.
Che et al. 2016 [28]	• Fast participant (high skill level)<br>• Slow participant (low skill level)	• The dyads achieve similar performance as faster participant's solo performance.<br>• Participants with similar skill level are more likely to improve their performance when paired.
Mireles et al. 2017 [6]	• Novice participant<br>• Expert participant	• Participants show greater improvement in task performance when connected with an expert.<br>• Participants connected with a novice partner show greater individual motor learning than participants connected with an expert.
Kager et al. 2019 [8]	• Novice participant<br>• Expert participant	• Participants connected with an expert show better task performance.<br>• Participants practiced with a novice partner show better motor learning outcomes.
Beckers et al. 2020 [37]	• More skilled participant<br>• Less skilled participant	• Compared to solo, dyadic performance improves only if the partner is better.<br>• Individual motor learning is higher when the partner is better
Mace et al. 2017 [33]	• Less-able participant<br>• More abled participant	• Performance of less-able subjects was enhanced by dual-player mode, by an amount proportionate to the partnership's mismatch<br>• The more abled partners' performances decreased by a similar amount.
Takagi et al. 2019 [12]	• One partner<br>• Two partner<br>• Three partner	• Interaction benefit on task performance improved as the group size increased<br>• Connecting to better partner(s) increased task performance<br>• Even if partner(s) skill level is slightly low, task performance improved compared to solo.
Beckers et al. 2018 [7]	• More skilled participant<br>• Less skilled participant	• Interacting with a better partner improves task performance compared to solo<br>• Interacting with a worse partner improves task performance compared to solo, albeit less than interaction with a better partner.
Takagi et al. 2018 [15]	• More skilled participant<br>• Less skilled participant	• Interacting with a better partner improves task performance more compared to interacting with a worse partner.
